# The vaginal bacteriome of pregnant Rwandan and Kenyan women, unique regional genera associations with preterm birth

**DOI:** 10.1186/s12866-025-04677-y

**Published:** 2026-02-05

**Authors:** Janet M. Wojcicki, Kilaza Samson Mwaikono, Etienne Nsereko, Nicole Santos, Linus W. Ndegwa, Wendy Blose, Shantelle Claasen-Weltz, Fadheela Patel, Samantha D. Africa, Julius Oyugi

**Affiliations:** 1 Dept of Pediatrics (GI,Hepatology and Nutrition), University of California, San Francisco, 550 16th Street, 4th floor, San Francisco, CA 94134-0136 USA; 2 Dar Es Salaam Institute of Technology, Dar Es Salaam, Tanzania; 3 College of Medicine and Health Sciences, University of Rwanda, Kigali, Rwanda; 4 Institue for Global Health Sciences, University of California, San Francisco, San Francisco, CA USA; 5 Kenya Medical Research Institute (KEMRI), Nairobi, Kenya; 6 Division of Medical Microbiology, University of Cape Town, Cape Town, South Africa; 7 Department of Medical Microbiology, Faculty of Health Sciences, University of Nairobi, Nairobi, Kenya

**Keywords:** Sub-Saharan Africa, Kenya, Rwanda, Preterm birth, Vaginal microbiota

## Abstract

**Background:**

Preterm birth, defined as gestational duration less than 37 weeks, often results in lifelong adverse health impacts for children. East African women have some of the highest rates of preterm birth globally. Previous studies have suggested that changes in the vaginal bacteriome may be associated with increased risk for preterm birth. The absence of *Lactobacilli* dominated vaginal flora and increased alpha and beta diversity have been associated with preterm birth in other contexts, primarily from US-based studies with African-American women. Few studies have been conducted with women from sub-Saharan Africa to assess whether vaginal bacteriome are associated with preterm birth risk.

**Methods:**

Using a longitudinal cohort study design, we recruited two groups of women from Kisumu, Kenya and Kigali, Rwanda in the first or second trimesters of pregnancy as confirmed via abdominal ultrasound. At the same time as abdominal ultrasound, vaginal bacteriome samples were collected and women were interviewed about sociodemographic and health backgrounds. Women were followed until delivery and gestational age was calculated and newborn birthweight determined. Sequencing of the 16S rRNA was subsequently conducted to assess specific vaginal flora and alpha and beta diversity by country and in relation to the preterm outcome. Pearson and Spearman correlation and heatmap of taxa associated with preterm versus term birth and by country of origin were calculated. Differences in genera abundance were assessed as well as metrics of alpha and beta diversity between Kenyan and Rwandan samples.

**Results:**

We included 118 women each from Kenya and Rwanda finding significant differences in beta diversity between countries but no association between alpha and beta diversity and preterm birth. We found differences in flora associated with preterm birth by country, although for both Kenya and Rwanda, higher counts of *Lactobacilli* were associated with term delivery. Different bacteriome determined preterm birth status by country with *Staphylococcus* and *Fannyhessea* significant in Rwanda but *Gardnerella * more common among Kenyan women.

**Conclusion:**

This is the first study that demonstrates there may be regional differences among East African women in the types of vaginal flora that are associated with preterm birth.

## Background

Preterm birth (PTB) is the second most common cause of neonatal death globally, and the most common cause of infant mortality in middle- and high-income economies [1, 2]. In the United States, population differences with respect to PTB exist, with women of African ancestry having a substantially larger burden of risk due to structural and social determinants of health including exposure to racism [3]. In sub-Saharan Africa, gene–environment interactions may play a more significant roles in determining the length of gestation with the bacteriome, playing a potentially critical role in predicting PTB [4].

### Sub-Saharan African studies and *Lactobacillus spp*

Studies from sub-Saharan Africa and other areas of the world indicate that vaginal colonization with *Lactobaccillus spp* is associated with reduced risk of vaginal infections, including sexually transmitted infections (STIs) and others associated with PTB such as bacterial vaginosis (BV). Other studies indicate that specific bacteria are associated with increased risk of PTB including the following bacteria with some overlap with bacteria associated with STIs: *Gardnerella vaginalis*,* Fannyhessea* (also known as *Atopobium*) and *Prevotella bivia* [5]. A study of Nigerian women with *Lactobacillus* dominant vaginal bacteriome were less likely to have risk factors associated with preterm birth including high risk human papilloma virus (HPV) [6] and *Gardnerella vaginalis* was associated with an absence of *Lactobacilli spp* and herpes simplex virus 2 (HSV-2) risk [7]. A study with a small, longitudinal Nigerian cohort found that women with *Lactobacillus spp* abundant vaginal bacteriome were more likely to deliver at a healthy gestation versus women lacking *Lactobacillus spp*,* as well as* those with *Fannyhessea* persistent throughout the pregnancy [8]. Another Nigerian study similarly found that women with *Fannyhessea*- dominated vaginal bacteriome in mid-pregnancy were more likely to have preterm birth outcomes [9]. In a study with HIV-positive women from Zambia, a novel *Gardnerella* metagenomic subspecies predicted spontaneous preterm birth [10].

Other studies suggest that among *Lactobacillus spp.* bacteriome, *Lactobacillus crispatus* promotes better vaginal health compared with *Lactobacillus iners* [11]. One explanation is that there is a paucity of cysteine transport mechanisms for *Lactobacillus iners* compared with other *Lactobacilli* [11]. Global studies in addition to Africa-specific ones suggest this finding; in a study with Chinese women with premature rupture of membranes (PPROM), *Lactobacillus iners* was more prevalent compared with *Lactobacillus crispatus* [12]. Studies from other areas of the world primarily among Caucasian women also suggest that a vaginal bacteriome with greater alpha and beta diversity increases risk for preterm birth although the mechanism has not been elucidated [13]. The small Nigerian cohort also found a higher amount of diversity among women with preterm birth [9].

Previous studies of East African women have found that there may be a higher colonization of bacteria associated with risk of preterm birth and this prevalence increases as women and girls age and become more sexually active. In a previous study of adult sexually active women from Kisumu, Kenya the vaginal taxa with the greatest relative abundance included *Gardnerella vaginalis* and *Lactobacillus iners* [7] both species and genera associated with greater risk for preterm birth. This differs from a study of younger adolescent Kenyan girls, also in Kisumu, where *Lactobacillus crispatus* had the highest relative abundance, a species known to be associated with healthy vaginal flora [14].

In this prospective, longitudinal study of vaginal bacteriome from two sites in East Africa, Kisumu, Kenya and Gasabo District, Kigali, Rwanda, we sought to assess whether there are any taxa associated with increased risk of preterm birth or earlier gestational age and whether genera known from previous studies to be associated with preterm birth were also associated with preterm birth in these East Africa contexts. We additionally evaluated whether there were any regional differences between the samples collected in Kenya versus Rwanda.

## Methods

### Study cohorts and sample selection

We enrolled women at two East African sites for this longitudinal study of microbial risk factors for preterm birth. In Kisumu, Kenya and Kigali Province, Rwanda, 537 and 420 women respectively between 18 and 49 years anticipating a healthy pregnancy were recruited into a cohort in 2019–2020 (Kenya) and 2017 (Rwanda). Women were assessed for singleton pregnancy as confirmed by abdominal ultrasound and were interviewed in language of choice (English, Kiswahili, Kiluo or Kinyrwanda) to collect socio-demographic and health associated data. Women were followed up through delivery to ascertain infant gestational age via telephone contact and medical record review.

Vaginal swabs were collected at the same time as abdominal ultrasound during clinic visits after the consent process at 9–15 weeks (Rwanda) and 22–24 weeks (Kenya) for a subset of the participants for subsequent bacteriome analysis [15]. Participants in both studies were confirmed negative from HIV and syphilis infections prior to enrollment. Participants who had abnormal vaginal discharge were treated for presumed vaginal infections in Kenya and after more specific testing in Rwanda [15]. A total of 120 and 121 samples from Kenya and Rwanda respectively were collected with oversampling of the preterm birth participants for the Kenya sample. The Rwandan samples were randomly selected from the overall cohort with the sample collection for bacteriome starting midway through the overall recruitment process with the sample size limited by financial constraints. This study was exploratory in nature and as such a sample size calculation was not conducted in relation to bacteriome differences. Vaginal swabs were collected by obstetricians during gynecological exams using the Norgen swab collection kit preserving DNA at room temperature (Thorold, ON Canada) until DNA extraction. Two vaginal swabs were collected from each study participant. The dataset for Rwanda included 547 taxonomic features (ASV; 2508282 reads) and for Kenya 370 taxonomic features (ASV; 2464333 reads). The Institutional Review Board at the University of Rwanda, Kenya Medical Research Institute (KEMRI) and the University of California, San Francisco approved these studies.

### DNA extraction

DNA was extracted from vaginal swabs stored in a DNA preservation medium (Thorold, ON, Canada) using the QIAsymphony^®^ SP instrument (Qiagen GmbH, Hilden, Germany) and the DSP Virus/Pathogen Mini Kit (catalogue no. 937036), following the manufacturer’s protocol. Mechanical lysis was performed off-board by bead beating in ZR BashingBead™ Lysis Tubes (Zymo Research Corp., Irvine, CA, United States) at 50 Hz for 5 min prior to automated extraction. The automated protocol includes enzymatic digestion with proteinase K to degrade bacterial cell walls and proteins. Extracted DNA was eluted in 60 µL. Negative and positive extraction controls were included in each run.

### Amplicon library preparation and illumina sequencing

The V4 region of the 16S rRNA gene was amplified using a two-step PCR protocol with primers and conditions previously described [16–18]. The first PCR amplified the target region, and the second PCR incorporated adapters and barcodes. Amplicons were purified using Agencourt^®^ AMPure^®^ XP beads, quantified, and pooled equimolarly. The final library was prepared and sequenced on the Illumina MiSeq platform (MiSeq Reagent Kit v3, 600-cycle) following the manufacturer’s instructions, including a 15% PhiX control spike-in [19, 20].

#### Bioinformatic and statistical analysis

The quality of raw sequences was assessed using the FASTQC tools [21, 22] while QIIME2 (Quantitative Insight into Microbial Ecology) suite of packages [23] was used for the upstream sequence analysis. The DADA2 (Divisive Amplicon Denoising Algorithms) plugin [24] was used for quality control and the taxonomic assignment was done using the feature-classifier classify-sklearn plugin [25] on the SILVA 138 reference pre-trained to V4 region of the 16S rRNA. Samples that had low quality sequences were filtered out. The merging of FASTQC files was done during importation into QIIME artefact at the start of the pipeline whereby the metadata information for each sample was used to distinguish between the two datasets. Statistical analysis was done using the Microbiome Analyst tool https://www.microbiomeanalyst.ca) [26]. Feature read count were normalized by total sum of squares (TSS) and square root transformed (Hellinger transformation to make the data comparable and easy to interpret [27]). The alpha diversity which included the Shannon Index, Simpson’s and Chao1 were estimated. Bray-Curtis and Jaccard distance metrics were used to generate the Principal Coordinate Analysis (PCoA) and non-metric distance (NMD) plots for beta diversity estimation of the bacterial community between preterm and term birth, as well as between Kenya and Rwanda. Alpha diversity measures within sample metrics including richness and evenness (diversity of the sample and number of distinct organisms) while beta diversity examines differences between samples/groups [28].

The Pearson and Spearman [29] correlation for parametric and non-parametric, respectively, and heatmap of taxa associated with preterm versus term birth and by country of origin were calculated. Network analysis was done to assess the association of bacterial community at genus, while random forest model [30] was used to identify individuals who had outlying vaginal bacteriome. Further, identification bacteria contributing to the outlying samples was performed. Descriptive maternal demographic and health information was provided in relation to country of origin and differences between the two countries was assessed statistically using chi-squared tests for categorical variables, Students t-tests and Wilcoxon rank sum for continuous ones.

Differences in abundance were assessed as well as metrics of alpha and beta diversity between Kenyan and Rwandan samples. Microbiome analyst (incorporating EdgeR and DESEQ2) was used for performing differential abundance analysis. Linear discriminant analysis (LDA) was used to test the differences in the abundance between groups, while permutational multivariate analysis of variance (permanova) was used for beta diversity analysis.

### Metabolic pathway analysis

Bacteriome functional prediction was done using Picrust2 tool [31]. Briefly, a FASTA file of a representative amplicons sequence variance (ASV) and a BIOM table of the abundance of each ASV generated on QIIME2 pipeline were used as input to the Picrust2. Placement of representative ASV into reference multiple sequence alignment was done using HMMER [32] and into reference phylogeny with EPA-NG [33], and GAPPA [34] for outputting new tree incorporating the ASV placement. Functional prediction was done using castor tool [35]; while pathway inference was done using MinPath tool [36]. Statistical analysis of predicted functional profiles was done using STAMP tool [37].

## Results

### Differences between Kenya and Rwandan women

We had 118 samples from Rwanda and Kenya respectively with corresponding demographics, newborn gestational age and health histories from each country. There were 3 (Rwanda) and 2 (Kenya) samples that were filtered out due to low quality sequences. Mean gestational age was 38.3 ± 2.2 weeks in Kenya and Rwanda combined with a lower gestational age in Kenyan infants (37.8 ± 2.4 weeks; *p* < 0.01: Table 1) with a corresponding higher percentage of Kenya infants preterm (28.0% versus 11.0% in Rwanda, *p* < 0.01). Birthweight was lower in the Rwandan samples (3128 ± 505 g versus 3353.3 ± 623.0 in Kenya, *p* < 0.01; Table 1). Notable demographics and health history differences in mothers from both countries included younger mothers in Kenya and more mothers married and in low income professions in Rwanda (Table 1). More mothers in Rwanda had no formal schooling (53.4% versus 0% in Kenya, *p* < 0.01; Table 1). Pre-pregnancy weight was higher among Kenyan mothers (64.1 ± 12.2kg versus 55.3 ± 14.1kg, *p* < 0.01; Table 1) as were previous preterm deliveries (26.3% versus 4.5%; *p* < 0.01 and Kenyan mothers had higher parity (2.5 ± 1.2 versus 1.5 ± 1.6, *p* < 0.01; Table 1). Rwandan mothers more often than Kenyan ones had abnormal vaginal discharge at study entry (31.4% versus 12.7%, *p* < 0.01; Table 1). Participants were asked about previous antibiotic exposure in relation to any dental procedure and none indicated any exposure (results not shown).

**Table 1:** Demographics and child delivery and maternal health specifics by country

	Total	Kenya	Rwanda	P-value
		(n=236)	(n=118)	(n=118)
		(Mean+/-SD or %)	(Mean +/-SD or %)	(Mean +/-SD or %)
Child factors				
Gestational age, wks	38.3±2.2	37.8±2.4	38.7±1.9	<0.01
Birthweight, g	3234.1±573.5	3353.3±623.0	3128.0±505.0	<0.01
Low birthweight	8.5%	8.6%	8.5%	0.98
Preterm delivery	19.5%	28.0%	11.0%	<0.01
Sex, male	50.5%	50.0%	50.9%	0.89
Maternal factors				
* Demographics*				
Age, years	27.4±5.8	26.4±5.8	28.4±5.7	<0.01
Educational level,				
Never schooled	26.7%	0%	53.4%	<0.01
Marital status, married	83.1%	71.2%	94.9%	<0.01
Low income profession	50.4%	37.3%	64.3%	<0.01
* Pregnancy and Reproductive Health*				
Pre-pregnancy wt, kg	58.9±14.0	64.1±12.2	55.3±14.1	<0.01
Previous preterm delivery	15.7%	26.3%	4.5%	<0.01
Parity	2.0±1.5	2.5±1.2	1.5±1.6	<0.01
Vaginal discharge present	22.0%	12.7%	31.3%	<0.01

### Differences between bacteriome samples by country

**Fig. 1:**
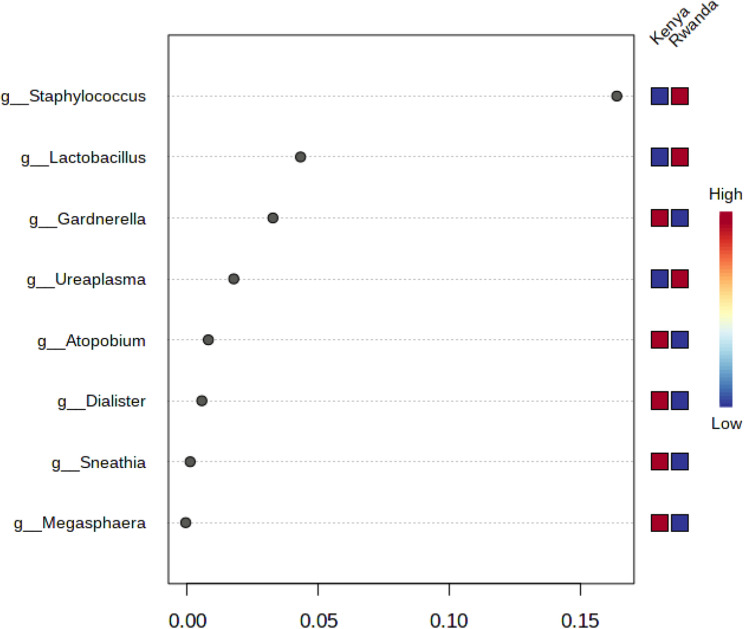
Differences in genera between Kenya and Rwandan samples. The y-axis, from top to bottom displays the bacterial genera ranked by their importance (mean decrease accuracy) for the country classification * g_ Atopobium is g_Fannyhessea in current nomenclature

**Fig. 2:**
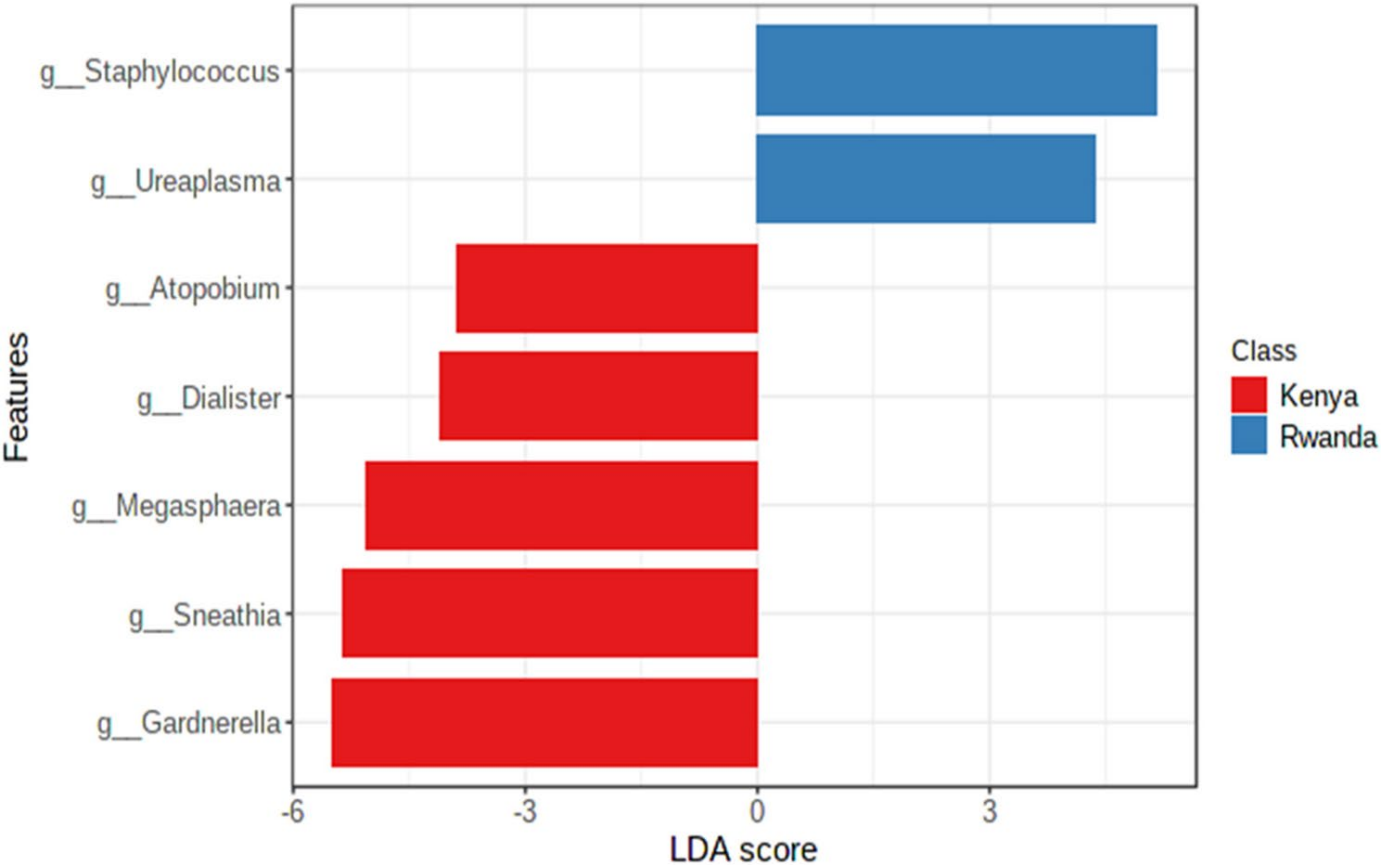
Linear discriminatory analysis plot showing association of vaginal bacteriome genera with Kenya and Rwanda *g_Atopobium is g_Fannyhessa in current nomenclature. Linear discriminant analysis score based on normalized abundance of data

**Fig. 3:**
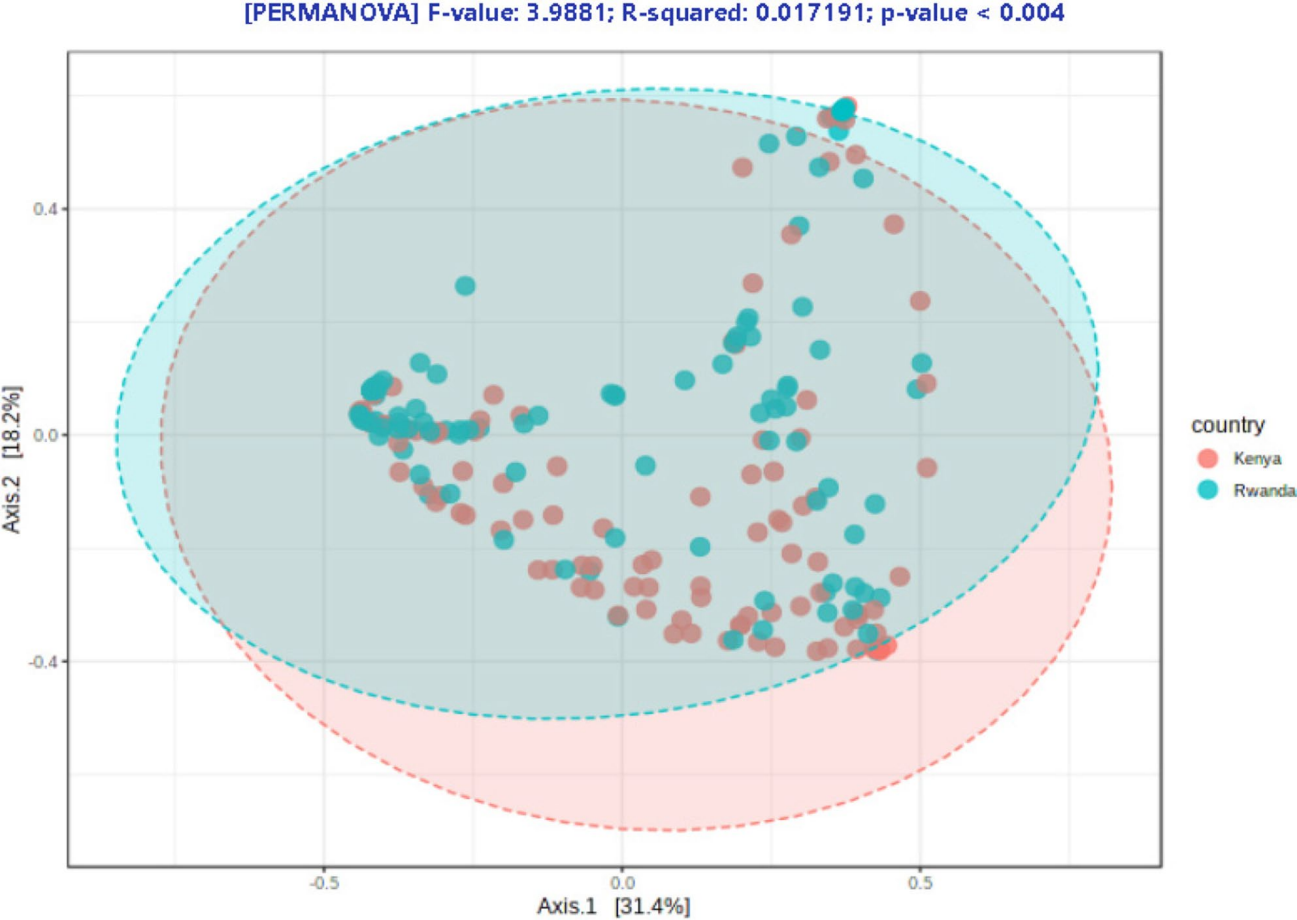
Beta diversity in Kenya and Rwanda vaginal bacteriome samples

Using difference metrics of alpha diversity (Chao1, Shannon and Simpson indexes), Kenya has higher levels of alpha diversity than Rwanda (Chao1, *p* = 0.0012; Shannon, *p* = 0.000138; Simpson, *p* = 0.000164). Differences in genera between countries included greater abundance of *Staphylococcus* (*p* = 7.93E-16), *Ureaplasma* (*p*= 7.50E-06) in Kenya samples and greater abundance of *Snethia* (p = 1.16E-07*)*, and *Dialister* (p = 2.63E-05) in Rwandan samples (Table 2). In a differential abundance analysis, *Staphylococcus* had the greatest importance (0.15) in determining Rwanda versus Kenya samples with *Lactobacillus* second at 0.05 with both genera higher in the Rwandan samples (Fig. 1). These values were higher and as such greater in there ability to discriminate between countries, or had less decrease in accuracy (compared with other genera). A greater linear discriminant analysis (LDA) score of greater than 4 for *Staphylococcus* and *Ureaplasma* (Kenya) and *Gardnerella*,* Sneathia*, *Megasphaera*, *Dialister and Fannyhessea* (Rwanda) less than − 4 suggest the difference in the abundance of these species by country (Fig. 2). The beta diversity results suggest that the microbial constitution differs significantly between the two cohorts (*p*< 0.01) with more beta diversity among Kenyan samples (Fig. 3).

**Table 2:** Differential abundance testing between Kenya and Rwanda (in order of significance)

Genus	log2FC^^^	FDR*	*P*-value
*Staphylococcus*	3.81	6.34E-15	7.93E-16
*Sneathia*	-2.07	4.64E-15	1.16E-07
*Ureaplasma*	1.79	2.00E-05	7.50E-06
*Dialister*	-1.10	5.27E-05	2.63E-05
*Fannyhessea*	1.56	0.000153	9.60E-05
*Megasphaera*	-1.52	0.000153	0.000115
*Gardnerella*	1.25	0.004213	0.003686
*Lactobacillus*	0.13	0.72616	0.71626

*False discovery rate

^Log two-fold change (Rwanda-Kenya)

### Differences by preterm birth status and possible confounders

We did not find any statistically significant differences in alpha or beta diversity by preterm birth/term (*p* = 0.99; *p* = 0.77 respectively) for the Rwandan dataset. Similarly, there were no statistically significant differences by preterm birth/term for the Kenyan dataset (*p* = 0.66; *p* = 0.48 for alpha and beta diversity respectively. We did find that women had abnormal vaginal discharge at entry had differences in alpha diversity (*p* < 0.01 for Cha1, Shannon, and Simpson indexes) but no differences in beta diversity.

#### Taxa associated with preterm birth and earlier gestational age

**Fig. 4:**
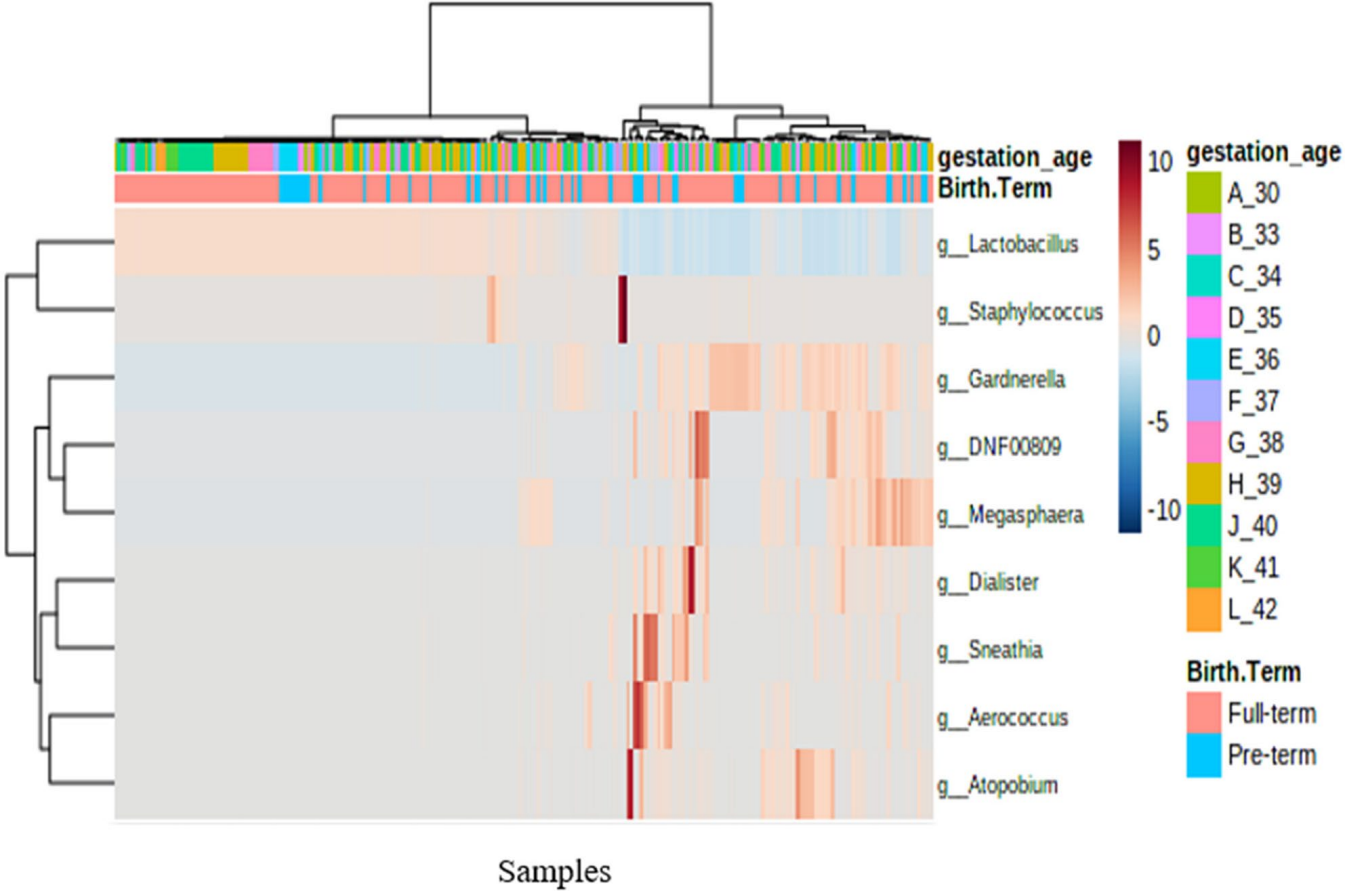
A clustered heat map showing variation of abundance in bacterial genera with regard to the gestation age and birth term for Rwanda and Kenya dataset *g_Atopobium is g_Fannyhessea as per latest nomenclature

In the combined groups from both countries, using clustered heat tree analysis, we found that when levels of *Lactobacillus* declined, *Staphylococcus*, *Sneathia*, *Gardnerella*,* Megasphaera*, *Dialister* and *Aerococcus* levels increased (Fig. 4). Specific genera were associated with earlier gestational age including *Aeroococcus* at a gestational age of 33 weeks (*p* = 3.45 E-19, FDR = 3.10 E-18) and *Sneathia* at 30 weeks and 37 weeks (*p* = 5.04E-19, FDR = 0.000151). Using heat tree analysis, genera *Sneathia* and in particular *Sneathia amnii* differentiated between 40 and 33 weeks gestational age (median difference 0.0267, *p* = 0.0168) and *Snethia annli* (mean difference = 0.02, *p* = 0.0198; Table 3). Comparing 40 with 34 weeks gestational age, significant genera differences included *Megasphaera* (*p* = 5.94E0.05) and *Aerococcus* (*p* = 0.03) with *Aerococccus christenseni* the species that was isolated differentiating these gestational time periods for the *Aerococcus* genera (*p* = 0.03; Table 3). Similar results were associated with a differential abundance analysis with mean decrease in accuracy in the model of 0.02 for *Sneathia* at 30 weeks and *Megasphaera* at 34 weeks (results not shown). *Lactobacillus* was also associated with a mean decrease in accuracy of -0.06 at 40 weeks (results not shown).

**Table 3:** Heat map results for differences between preterm and term based on gestational age

Genera/Taxa	Gestational Age		
	Comparison Time	Mean Difference	*P* value
*Sneathia*	33 versus 40	0.02677	0.0168
*Sneathia amnii*	33 versus 40	0.022165	0.0198
*Megasphaera*	34 versus 39	-0.0779	0.00093
*Megapshaera*	40 versus 34	-0.0919	5.94E-05
*Fannyhessea*	34 versus 39	-0.017	0.045
*Aerococcus*	34 versus 39	-0.001	0.006
*Aerococcus christensenii*	40 versus 34	-0.009	0.0371

**Fig. 5:**
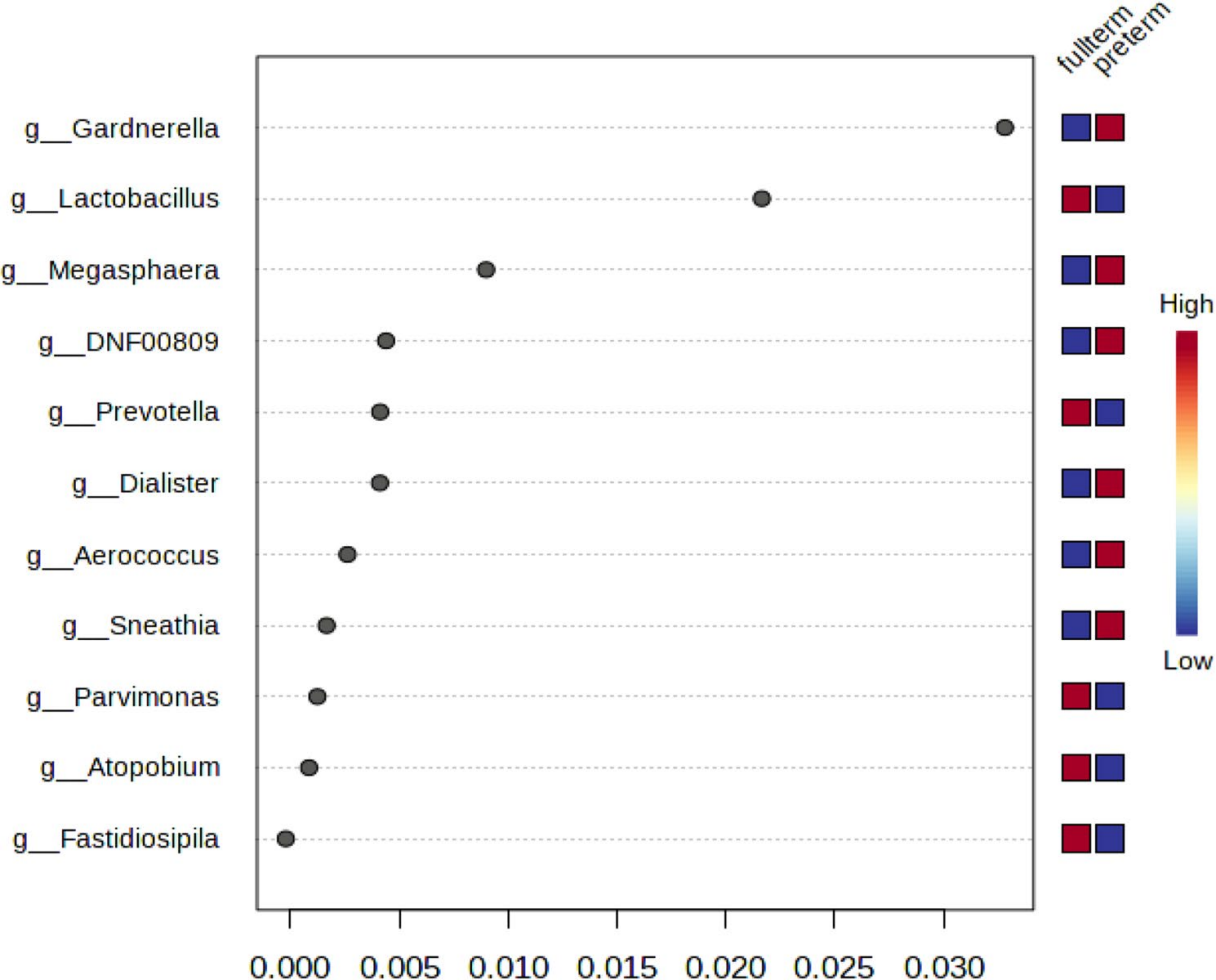
Kenya: differences between full-term and preterm in vaginal bacteriome (Genera). The y-axis, from top to bottom, displays the genera ranked by their importance (mean decrease in accuracy) for the birth term classification * g_ Atopobium is g_Fannyhessea in current nomenclature

**Fig. 6:**
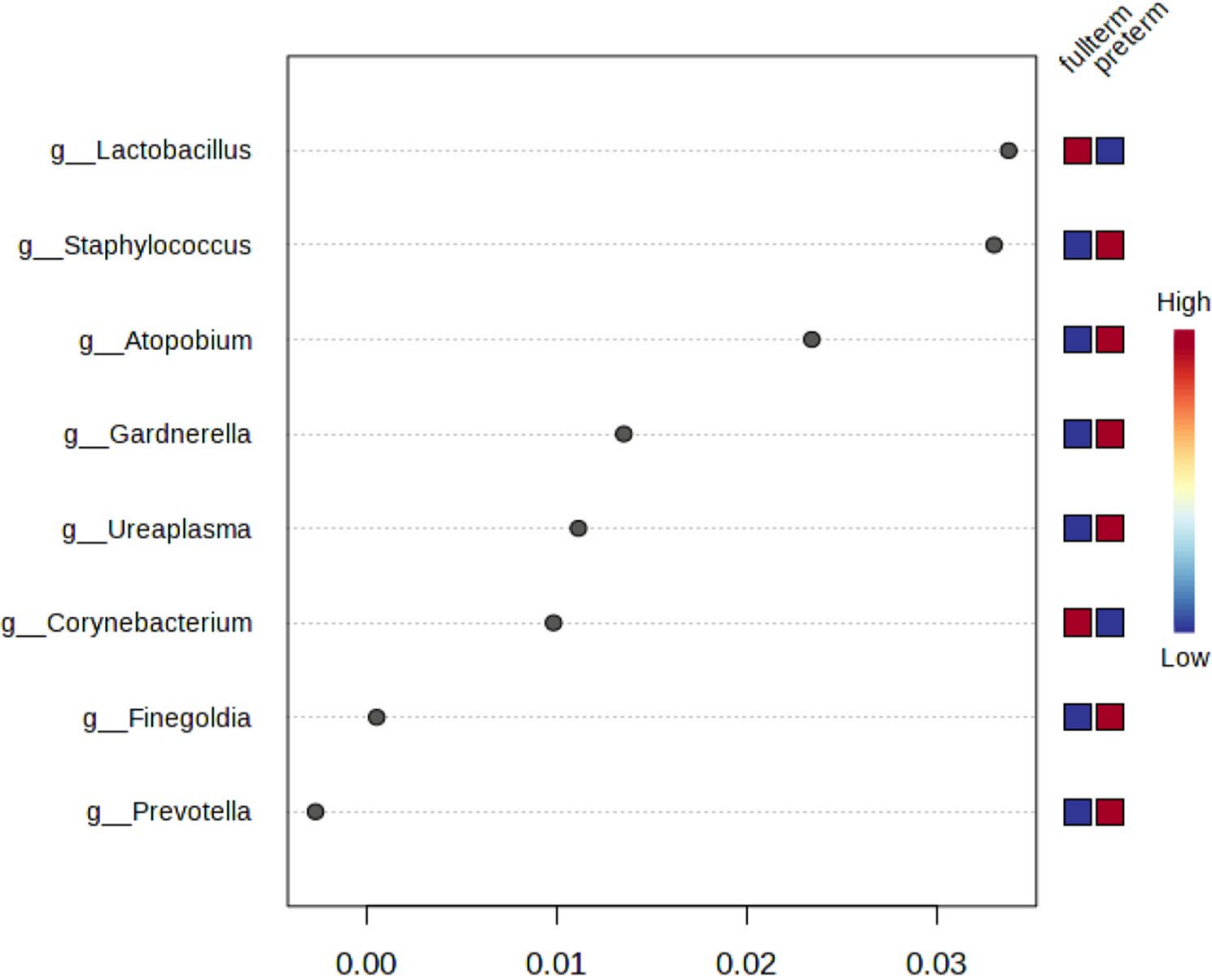
Rwanda: differences between full-term and preterm in vaginal bacteriome (Genera). The y-axis, from top to bottom displays the bacterial genera ranked by their importance m decrease in accuracy) for the birth term classification * g_ Atopobium is g_Fannyhessea in current nomenclature

We also examined which organisms differentiated term versus preterm by country. *Gardnerella* differentiated the Kenyan samples between preterm and term with a higher abundance in preterm birth (mean decreased in accuracy for the model, 0.03) versus *Lactobacillus* in higher abundance in term infants (mean decrease in accuracy, 0.02) (Fig. 5) compared with Rwanda where we found that *Staphylococcus* and *Fannyhessea* were associated with preterm birth (mean decrease in accuracy 0.03 and 0.02 respectively) (Fig. 6). *Lactobacillus* was similarly associated with term birth for Rwandan samples with a mean decrease in accuracy of 0.03 (Fig. 6). For the early preterm (gestational age of 30 weeks), for Kenyan samples, *Sneathia* and *Parvimonas* were in higher counts.

### Differences between Rwanda and Kenya for earlier gestational age in functional pathways

We found significant differences between functional pathways for term versus preterm births comparing Kenya with Rwanda. In Kenya specifically, we found a significant mean difference in the super pathway of guanosine nucleotides de novo biosynthesis II (*p* = 0.01; mean difference 2519 ± 1254), pyrimidine deoxyribonucleotides de novo biosynthesis I (*p* = 0.011; mean difference 1991 ± 904) and pyrimidine deoxyribonucleotide phosphorylation (*p* = 0.013; mean difference 2023 ± 861). By contrast, for the Rwanda samples we found a difference in glycerol degradation to butanol pathway for the term versus preterm births (*p* < 0.01; mean difference 11 ± 59).

## Discussion

Our study provides a comparison of the bacteriome found in term and preterm births comparing Rwandan and Kenyan women using a longitudinal study design. Our study used a similar process of inclusion and exclusion criteria to recruit participants although Rwandan mothers were recruited at an earlier gestational age than Kenyan ones. Some findings were common to both countries and sample populations including a higher abundance of *Lactobacillus* among term pregnancies in Kenya and Rwanda similar to studies from other parts of sub-Saharan Africa [8]. There were also significant differences between the two countries with greater abundance of *Gardernella* in Kenya preterm deliveries similar to other Kenyan studies from Kisumu [7] and Nairobi [38] versus *Staphylococcus* and *Fannyhessea* in Rwandan preterm deliveries. To our knowledge, this is the first study to assess the vaginal bacteriome among Rwandan women in relation to preterm birth.

Independent of gestational age at delivery, there were significant differences in the vaginal bacterial composition of Kenyan versus Rwandan samples with overall higher counts of *Staphylococcus* and *Lactobacillus* among Rwandan women and higher overall alpha diversity among the Kenyan samples. Other studies have noted that there are important differences in the bacteriome between ethnic and racial groups in the United States and globally between populations, attributing some of these differences to host genetic factors including the innate or adaptive immune system [39]. The differences observed between Kenyan and Rwandan bacteriome constitutions could reflect environmental or cultural factors that impact vaginal bacteriome, host genetic factors or alternatively sampling biases between the two areas. We did not adjust for any sociodemographic differences between the population groups including maternal age or education level which could have impacted findings; however, there are significant population-level differences in socioeconomics between the two countries with literacy rates, education status and income higher in Kenya than Rwanda [40]. Additionally, the Rwandan women were recruited at an earlier gestational age (9–15 weeks) than their Kenyan counterparts (22–24 weeks) which could explain some differences in bacterial composition. However, previous studies have found a greater abundance of *Lactobacillus* dominated vaginal bacterial compositions in the 2nd versus 1st trimesters and a decrease in alpha diversity later in pregnancy contrary to our findings [41].

### Preterm births

Our findings of higher counts of *Fannyhessea* in preterm Rwandan pregnancies was similar to the only other longitudinal study to our knowledge of bacteriome in pregnant sub-Saharan African women that also found higher counts of *Fannyhessea* in preterm deliveries. A study of pregnant Nigerian women [8, 9] found higher counts of *Fannyhessea* among women with preterm deliveries (mean of *A. vaginae* of 0.446 versus 0.046 for preterm versus term births). Other studies including European ones have found higher counts of *Fannyhessea* and *Gardnerella* in women with preterm delivery [42]. These bacteria can create a more anaerobic condition and could replace lactic acid bacteria producing species [43]; *G. vaginalis* may create a cytotoxic environment and adheres to vaginal epithelial cells according to previous studies [44]. Preterm birth studies with a high percentage of African-American women in the United States found high levels of *G. vaginalis* in preterm births and lower levels of *Lactobacillus*, similar to our findings from Kenya [45]. Another study from the Czech republic similarly found a high percentage of *G. vaginalis* in women with PPROM (94%); *n* = 379/405) [43].

Similar to other studies that have noted that a *Lactobacillus* dominated bacteriome is associated with improved maternal outcomes, a high concentration of *Lactobacillus* was observed in higher counts in bacteriomes in both Kenyan and Rwandan women in our study who delivered term infants [46]. *Lactobacillus* spp. may inhibit growth of pathogenic bacterial species by creating an acidic vaginal microenvironment. Some species of *Lactobacillus* produce bacteriocins and hydrogen peroxide which suppress other bacterial organisms [47]. Previous studies in African women from Nigeria found a higher count specifically of *L. iners* in those women delivering term versus preterm infants [9]. We, however, did not differentiate between *Lactobacilli* bacteria at the level of the species between preterm and term births which was one of the limitations of our study. The Nigerian study also found a higher alpha diversity among mothers that had preterm delivery in contrast with our findings but also confirms previous studies of greater alpha diversity among the vaginal bacteriome of preterm births [48].

We also examined which organisms differentiated term versus preterm by country. *Gardnerella* differentiated the Kenyan samples between preterm and term with a higher abundance in preterm birth versus *Lactobacillus* in higher abundance in term infants. For Rwanda, we found that *Staphylococcus* and *Fannyhessea* were associated with preterm birth. *Lactobacillus* was similarly associated with term birth for Rwandan samples. For the early preterm (gestational age of 30 weeks), for Kenyan samples, *Sneathia* and *Parvimonas* were in higher counts. *Snethia* and *Fannyhessea* were similarly observed in greater abundance among South African women with a heightened risk of human papillomavirus (HPV) infection. Previous studies with healthy African-American women and African-American women with BV have found a greater likelihood of colonization with *Sneathia* compared with healthy women of European ancestry and those without BV [47]. *S. aureus* has also previously been described as risk factor for chorioamnionitis and preterm birth [49]. It is not clear why preterm birth is associated with *Staphylococcus* and *Fannyhessea* in Rwanda but *Gardnerella* among Kenyan women.

In Kenya specifically, we found a significant mean difference in the super pathway of guanosine nucleotides de novo biosynthesis II, pyrimidine deoxyribonucleotides de novo biosynthesis I and pyrimidine deoxyribonucleotide phosphorylation. By contrast, for the Rwanda samples we found a difference in glycerol degradation to butanol pathway.

### Metabolic pathways

Other African studies have found that the nonoxidative branch of the pentose pathway enriched in preterm deliveries compared to term ones [9]. Potentially a high metabolic activity of this gene may promote proinflammatory cytokines, particularly with immune cell activation resulting in reactive oxygen species production through NADPH. Within our Rwandan samples, an elevated glycerol to butanol pathway was identified in association with preterm birth. Other studies have linked this pathway to elevated inflammation and allergic conditions [50]. The differences in the metabolic pathways for our Kenyan samples have not been previously described in preterm or gestational duration studies including the guanosine nucleotides de novo biosynthesis, pyrimidine ribonucleotides de novo biosynthesis and pyrimidine deoxyribonucleotides de novo biosynthesis and phosphorylation pathways.

### Alpha and beta diversity

The overall alpha diversity levels among our populations from Kenya and Rwanda compared similarly with data from African diaspora populations described by Fettweis et al., (2014) (via Simpson’s Inverse) 2.26 ± 3.64 for Kenya and 3.45 ± 4.37 for Rwanda and similar to the 2.7 ± 1.9 reported by Fettweis et al., 2014 and higher than European origin populations. (1.8 ± 1.1). The levels of beta diversity that we had in both populations were 1.08 ± 4.59 for Kenya and 1.08 ± 4.67 for Rwanda (using Bray-Curtis) was higher than that reported previously with African-American and European populations [45] (0.69 ± 0.23 and 0.79 ± 20.23 respectively).

Previous studies have suggested that higher alpha diversity scores for vaginal bacteriome with greater diversity are associated with vaginal infections including BV and preterm birth [51]. We did not find any differences between preterm and term births and alpha and beta diversity by country in contrast with other studies that have found more diversity with preterm birth [46, 48]. However, the majority of these studies have been with other population groups, outside of sub-Saharan Africa including Caucasian only population groups [13]. Other African studies have not found differences in alpha or beta diversity for risk factors of preterm birth including vaginal infections [38, 52] nor have other studies with African-American populations [53] with the exception of the longitudinal cohort study from Nigeria [8]. It is possible that the overall greater microbial diversity found in African and African diaspora populations necessitates a larger sample size to determine changes in diversity or that microbial diversity may be less significant in African populations. Some of these other studies have also assessed vaginal samples during the delivery or postpartum period; microbial profiles are notably different in pregnancy than the postpartum period [9].

### Conclusions and limitations

Ours is the first longitudinal sub-Saharan African study to assess differences in vaginal microbial profiles between two population groups using a similar study design. Many previous studies with African women have been conducted in the context of HIV [10] or with women engaged in sex work [54]. We found significant differences in bacteria present mid pregnancy associated with risk of preterm birth by country status (*Staphylococcus* and *Fannyhessea* significant in Rwanda but *Gardnerella* among Kenyan women). We also found similar to other studies that *Lactobacilli* were increased in the samples of women who delivered term infants. In contrast with other studies, we did not find any differences in alpha or beta diversity comparing the vaginal bacteriome of women who delivered preterm versus term infants. While we had a relatively large sample size compared to other studies, we did not assess bacteriome differences by maternal health including pre-existing STIs or demographic factors and some of these variables could have confounded the relationship between the vaginal bacteriome and preterm birth. We also did not assess lifetime history of antibiotic use among participants, although we did survey participants regarding antibiotic use in relation to previous dental procedures. Additionally, there may have been regional differences between Kenyan and Rwanda in terms of vaginal washing/drying or other intravaginal practices may have impacted bacteriome findings [55, 56]. Lastly, we did not collect any information on dietary practices which could have impacted bacteriome and partially explained regional differences. Future studies are needed with more diverse African populations to better understand the role of maternal age, health and demographic for bacteriome outcomes.

## Data Availability

Data and materials are available on request from the corresponding author.

## Abbreviations

ACE: Abundance based coverage estimator
DNA: Deoxyribonucleic acid
PCR: Polymerase chain reaction
HIV: Human immunodeficiency virus
NMA: Non–metric analysis
PCoA: Principal coordinate analysis
PTB: Preterm birth
TSS: Total sum of squares
